# Development of a rapid and highly sensitive nucleic acid-based diagnostic test for schistosomes, leveraging on identical multi-repeat sequences

**DOI:** 10.3389/fpara.2024.1361493

**Published:** 2024-03-14

**Authors:** Ombeni Ally, Bernard N. Kanoi, Shwetha Kamath, Clement Shiluli, Eric M. Ndombi, Maurice Odiere, Gerald Misinzo, Steven Ger Nyanjom, Chunduri Kiran Kumar, Lucy Ochola, Srinivasa Raju Lolabattu, Jesse Gitaka

**Affiliations:** ^1^ Department of Molecular Biology and Biotechnology, Pan African University Institute for Basic Sciences, Technology and Innovation (PAUSTI), Nairobi, Kenya; ^2^ Department of Biology, College of Natural and Mathematical Sciences, University of Dodoma, Dodoma, Tanzania; ^3^ Center for Research in Infectious Diseases, College of Graduate Studies and Research, Mount Kenya University, Thika, Kenya; ^4^ Division of Research and Development, Jigsaw Bio Solutions Private Limited, Bangalore, India; ^5^ Centre for Global Health Research, Kenya Medical Research Institute, Kisumu, Kenya; ^6^ Department of Medical Microbiology and Parasitology, Kenyatta University, Nairobi, Kenya; ^7^ SACIDS Africa Center of Excellence for Infectious Diseases, Sokoine University of Agriculture, Morogoro, Tanzania; ^8^ Department of Biochemistry, Jomo Kenyatta University of Agriculture and Technology, Nairobi, Kenya; ^9^ Department of Computer Science and Applications, KL University, Andhra Pradesh, Guntur, India; ^10^ Department of Tropical and Infectious Diseases, Institute of Primate Research, Nairobi, Kenya

**Keywords:** *Schistosoma mansoni*, *Schistosoma haematobium*, schistosomiasis, PCR, qPCR, IMRS

## Abstract

**Introduction:**

Schistosomiasis (Bilharzia), a neglected tropical disease caused by *Schistosoma* parasites, afflicts over 240 million people globally, disproportionately impacting Sub-Saharan Africa. Current diagnostic tests, despite their utility, suffer from limitations like low sensitivity. Polymerase chain reaction (PCR) and quantitative real-time PCR (qPCR) remain the most common and sensitive nucleic acid amplification tests. Still, the sensitivity of nucleic acid amplification tests is significantly affected by the copy number of amplification targets, resulting in underestimation of true *Schistosoma* infections, especially in low transmission settings. Additionally, lengthy qPCR run times pose challenges when dealing with large sample volumes and limited resources. In this study, the identical multi-repeat sequences (IMRS) were used as a novel approach to enhance the sensitivity of nucleic acid-based Bilharzia diagnosis.

**Methods:**

To identify novel genomic repeat regions, we utilized the IMRS algorithm, with modifications to enable larger target region (100-200bp) identification instead of smaller sequences (18-30bp). These regions enabled customised primer-probe design to suit requirements for qPCR assay. To lower the qPCR amplification times, the assay was conducted using fast cycling condition. Regression analysis, and qPCR data visualization was conducted using Python programming.

**Results:**

Using *Schistosoma mansoni* and *S. haematobium*, we found that IMRS-based qPCR, employing genus-specific primers and TaqMan probes, offers exceptional analytical sensitivity, detecting as little as a single genome copy per microliter within 36 minutes.

**Discussion:**

The lowest concentration of DNA detected using IMRS-based PCR and qPCR represented tenfold improvement over conventional PCR. As part of further development, there is a need to compare IMRS-based qPCR against other qPCR methods for *Schistosoma* spp. Nonetheless, IMRS-based diagnostics promise a significant advancement in bilharzia diagnosis, particularly in low-transmission settings, potentially facilitating more effective control and treatment strategies.

## Introduction

Bilharzia (schistosomiasis) is the second most devastating parasitic disease after malaria, affecting over 240 million people worldwide (World Health Organization [WHO], 2022a), of which 90% reside in Africa (Aula et al., 2021). *Schistosoma mansoni* and *S. haematobium* are the significant causes of schistosomiasis, resulting to intestinal and urogenital schistosomiasis, respectively (Aula et al., 2021). Intestinal schistosomiasis causes diarrhea, abdominal pain, bloody stool, and hepatosplenomegaly in advanced cases, and urogenital schistosomiasis manifests through the genital and urinary system, resulting in haematuria and bladder cancer when the disease is left untreated for a prolonged period (Yohana et al., 2023).

Despite the absence of an approved vaccine against schistosomiasis to date, praziquantel still remains the most widely used control measure for schistosomes (World Health Organization [WHO], 2022a; Kokaliaris et al., 2022), imposing the need for alternative species-specific and genus-specific diagnostic approaches, primarily for *Schistosoma* species-specific surveillance and elimination purposes, respectively. To date, despite its low sensitivity, microscopy remains the gold standard approach for diagnosing schistosomiasis, and this approach offers high specificity compared to the existing diagnostic approaches. The WHO’s recommendation on the use of microscopy Kato-Katz (KK) and urine examination is restricted to individuals with moderate to high parasite loads for consistent results. In individual with low parasite load, the number of schistosome eggs in stool and urine decreases, making it difficult to detect by microscopic KK or urine examination. Alternative methods have been evaluated including circulating cathodic antigen (CCA), the most common point-of care assay for diagnosing schistosomiasis, and the commercial version is available for field diagnosis (Hoekstra et al., 2021; De Dood et al., 2018). However, research findings and WHO reports have documented the significant bias of the CCA test for *S. mansoni* over other species and further batch-to-batch variation makes it difficult to accurately estimate the actual prevalence using CCA test, especially in low infectious settings (Hoekstra et al., 2021; World Health Organization [WHO], 2021). In a study by Graeff-Teixeira et al. (2021), for instance, POC-CCA resulted in 62.1% specificity, but when the samples were tested using two different batches and after being stored at -20°C, the specificities for the two batches were 34.3% and 75.0%, respectively. Herein, the weak agreement was an indicator of the urgent need for the standardization of the manufacturing process of the POC-CCA tests. Straily et al. (2022), as well, found that the POC-CCA assay often gave false negative results for samples that KK had confirmed to be positive. In their study, the authors reported that 356 KK-confirmed samples were designated as negative by POC-CCA test, representing a 2.8% false negative rate for all tested samples, with 326 (91.6%) of these samples originating from low-intensity individuals.

Nucleic acid amplification tests (NAATs) are also proposed to offer good sensitivity than microscopy, and real‐time polymerase chain reaction (qPCR), loop mediated isothermal amplification (LAMP) and conventional PCR (cPCR) are most common approaches for surveillance of schistosomes (Fernández-Soto et al., 2020; Weerakoon et al., 2015; Chala, 2023). Still, the sensitivity of NAATs can be significantly affected by the copy number of amplification targets (Raju et al., 2019), resulting in an underestimation of true *Schistosoma* infections (Hoekstra et al., 2022; Keller et al., 2020), especially in low transmission settings. The WHO, through its guidelines for control and elimination of human schistosomiasis, published online in 2022 (World Health Organization [WHO], 2022b), pointed that there is the need for further evaluation of sensitivity of NAATs, as little is known on their performance in comparison to the gold standard, KK and urine examination. Few studies however, have indicated the possibility of some NAATs, including qPCR, to designate negative to some microscopy-confirmed positive samples, particularly in low infectious burden (Hoekstra et al., 2022; Meurs et al., 2015; Feleke et al., 2023).

One study found that qPCR targeting the Dra1 repeat unit in *S. haematobium* had overall sensitivity of 89.5%, and 82.8% specificity, and the author reported that the assay missed 11 out of 105 urine examined positive samples, while the sensitivity increased to 96.5%, when samples with ≥50 eggs per gram (epg) were re-evaluated (Keller et al., 2020). Another study reported the ITS2 qPCR to miss some positive samples, and the author found that the sensitivity ranged from 79% to 87% for low infection burden (1-99 epg), 83% to 97% for moderate infection (100-399 epg), and 100% for heavy infection (≥400 epg) (Meurs et al., 2015). Hoekstra et al. (2022) and Armoo et al. (2020) reported a reduced sensitivity for qPCR compared to Up-Converting reporter Particle technology based, Lateral Flow (UCP-LF) CAA test and the urine-CCA assay, respectively. Unlike the CCA test, which is WHO-recommended for *S. mansoni* detection, the CAA test is reported to offer much better sensitivity against *S. haematobium* (De Dood et al., 2018; Hoekstra et al., 2022; Fasogbon et al., 2023). When Hoekstra et al., 2022 looked at the performance of the CAA test, urine microscopy, and PCR test, the authors found that more people were testing positive in light infections individual when UCP-CAA test was used instead of PCR (Hoekstra et al., 2022). Still, further studies are needed to validate and contextualize the use of the CAA test at POC settings (Fasogbon et al., 2023).

Isothermal amplification assays have also indicated comparable to much more sensitivity than the cPCR and qPCR (MacGregor et al., 2023; García-Bernalt Diego et al., 2021). For example, when LAMP was compared to other tests, findings indicated its better performance in sensitivity compared to nested PCR and KK in snail and human samples, respectively (Gandasegui et al., 2018). WHO (2022), however, adapted the findings from a systematic review and meta-analysis articles, on the performance of two LAMP assays, one with 87% sensitivity and 50% specificity against KK and the other with 66% sensitivity and 79% specificity based on the urine examination results (World Health Organization [WHO], 2022b). In this instance, the relatively low to moderate specificity may have resulted from the fact that microscopy is less sensitive, but the issue of low to moderate sensitivity of LAMP against samples with *Schistosoma* eggs needs attention to lower the possibility of false negatives. The need for a single temperature and shorter amplification times, most takes approximately 30 minutes (García-Bernalt Diego et al., 2021), gives isothermal amplification assays more potential to replace NAATs that need expensive machines (MacGregor et al., 2023; Gandasegui et al., 2015). However, the need for multiple pairs of primers complicates the initial optimization, and the reported high chance of carry-over contamination raises the risk of false positives in LAMP reactions (Quyen et al., 2022; Zen et al., 2020), which necessitates the need for cPCR and qPCR, especially in well-established laboratories with trained staff.

On the other hand, lengthy qPCR run times pose challenges when dealing with large sample volumes and limited resources. In most cases, a standard qPCR run takes approximately 45 to 120 minutes to complete (Nayab et al., 2008; Bustin and Nolan, 2020), and this includes the time taken for initial denaturation to activate the modified hot-start DNA polymerase, usually followed by 30-50 cycles of denaturation, annealing, and extension (Nayab et al., 2008; Aryeetey et al., 2013). Despite the enormous advances in qPCR machines and improved polymerization speed and processivity of polymerases, most existing qPCR assays takes approximately one hour on average (Bustin and Nolan, 2020). 

In developing genus-specific NAATs, the primary concern is the availability of a shared nucleotide sequence among the pathogens or parasites of interest. Schistosomes, however, have a relatively large genome size, with about 409.57 megabase (mb) (49.23%) repeat contents and 400.27 mb (54.57%) repeat contents in *S. mansoni* and *S. haematobium*, respectively, and most nucleotide sequences are shared among the two species (Stroehlein et al., 2022). With the advances in genome-mining approaches that allow the identification of the repeated sequences in genomes, it is possible to improve the sensitivity of the NAATs-based genus-specific assay for diagnosing schistosomiasis, targeting the repeated sequences in schistosomes. In addition, the availability of modified hot-start polymerases, requiring 1 to 2 minutes activation time, and the enhanced performance speed ensures the reduction of qPCR run time without affecting the assay’s sensitivity.

To improve sensitivity and specificity of molecular diagnostic assays, Raju et al. (2019) proposed novel genome mining approach using the IMRS algorithm to identify new, pathogen-specific biomarker target regions called IMRS and designed nucleic acid-amplification assays using IMRS as primers. The IMRS initiate amplification from multiple loci across the genome improving overall analytical sensitivity. The IMRS platform technology was used to design ultrasensitive qPCR (Raju et al., 2019), iso-IMRS isothermal amplification assay for *Plasmodium falciparum* malaria (Kolluri et al., 2021) and other commercial TaqMan IVD assays (unpublished). In this study, we have used the IMRS genome mining approach to identify all possible repeat regions in the genomes of *S. mansoni* and *S. haematobium* and designed TaqMan qPCR assay by using best ranked IMRS region. This assay was 10-fold more sensitive than conventional assay targeting 16S rRNA for simultaneous detection of *S. mansoni* and *S. haematobium* with a time-to-result of 36 minutes. Herein, we report our findings.

## Materials and methods

### Control samples

The standard genomic DNA isolated from pooled adult male and female *S. mansoni*, strain NMRI (NR-28910) and adult male and female *S. haematobium*, Egyptian Strain (NR-31682) were obtained from the Biodefence and Emerging Infectious Research Resources (ATCC, Virginia, United States). *S. mansoni* miracidia DNA from pooled human fecal samples and positive and negative DNA of schistosomes from field snails were obtained from Kenya Medical Research Institute (KEMRI), Kisumu, Kenya. DNA samples from two Chronic *S. mansoni-*infected Olive baboons maintained at the Institute of Primate Research (IPR) Animal sciences department were provided by the Department of Infectious Diseases at the IPR, Nairobi, Kenya, and *Ancylostoma lumbricoides* positive DNA samples were obtained from Jomo Kenyatta University of Agriculture and Technology (JKUAT), Nairobi, Kenya (a kind donation from Mr Jackan Moshe).

### Genome mining for IMRS

To identify novel genomic repeat regions, we utilized the IMRS algorithm (Raju et al., 2019; Kolluri et al., 2021), with modifications to enable larger target region (100-200bp) identification instead of smaller sequences (18-30bp). These regions enabled customized primer-probe design to suit assay requirements. Briefly, genome assembly sequences for *S. mansoni, S. haematobium* were used as input to the IMRS algorithm to identify identical, repetitive sequence substrings from multiple loci within the genome sequences. A library of unique repeat sequences was obtained as output that were further filtered for sequences that were having a maximum number of repeats. The best ranked repeat region of 151 bp (5’-AAGGTCGGATCTACAACGCGTCGGTGAGAGCAGTTTTGCTCTATGCTTGTGAAACCTGGCCTCTCCGAGTTGAGGATGTTAGACGTCTCTCTGTGTTCGATCATCGTTGTCTCCGAAGGATTGCTGACATCCAGTGGCAACACCATGTTAG-3’) was predicted to amplify the largest amount of DNA, leading to the best possible analytical sensitivity, and hence was chosen for this study. This region was evaluated using the NIH’s Basic Local Alignment Search Tool (BLAST) to ensure that it was specific to the *Schistosoma* genomes. Forward primer-1 (5’-AAGGTCGGATCTACAACGCGTC-3’) and reverse primer-1 (5’-CTAACATGGTGTTGCCACTGGAT-3’) were selected from this region for IMRS-based cPCR, targeting the entire 151 bp amplicon.

### Primers and probe for IMRS qPCR

Further primer-probe optimization to meet qPCR assay requirements was done. The chosen 151 bp IMRS output region sequence was used as an input in the PrimerQuest Tool of the OligoAnalyzer™ Tool from IDT, to design forward primer, probe and reverse primer and forward primer-2; (CGTCGGTGAGAGCAGTTT): reverse primer-2; (CCTTCGGAGACAACGATGAT) and probe sequence; (FAM-TATGCTTGTGAAACCTGGCCTCTCC-BHQ-1) were selected for the amplification of the targeted 101 bp in schistosomes.

### Circa plots

**Figure 1:**
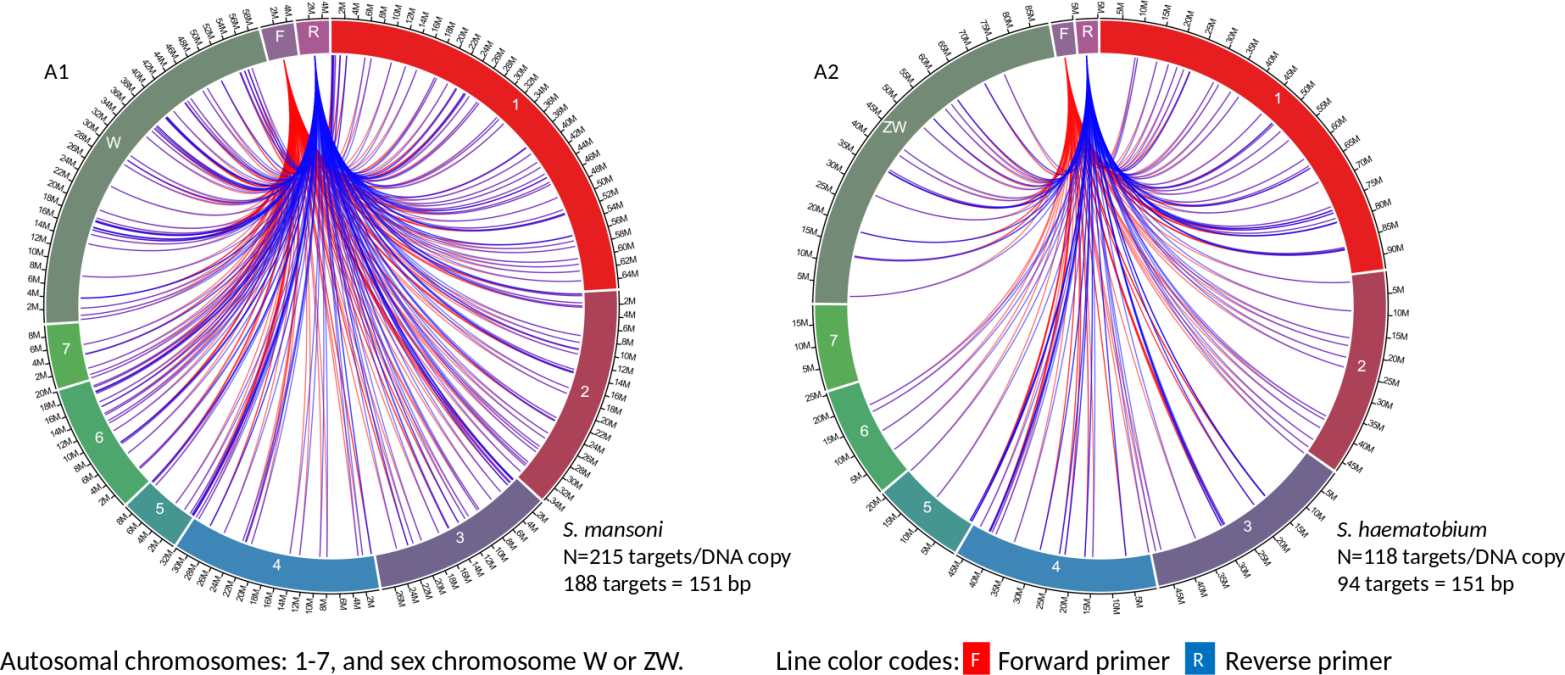
The circa plot for the distribution of IMRS primers and probe on schistosomes. **(A1, A2)** shows the distribution of forward primer 1, reverse primer 1 on *S. mansoni* and *S. haematobium*, respectively. The number outside the chromosomes represents the chromosome size in Megabase pair (M).

**Figure 2:**
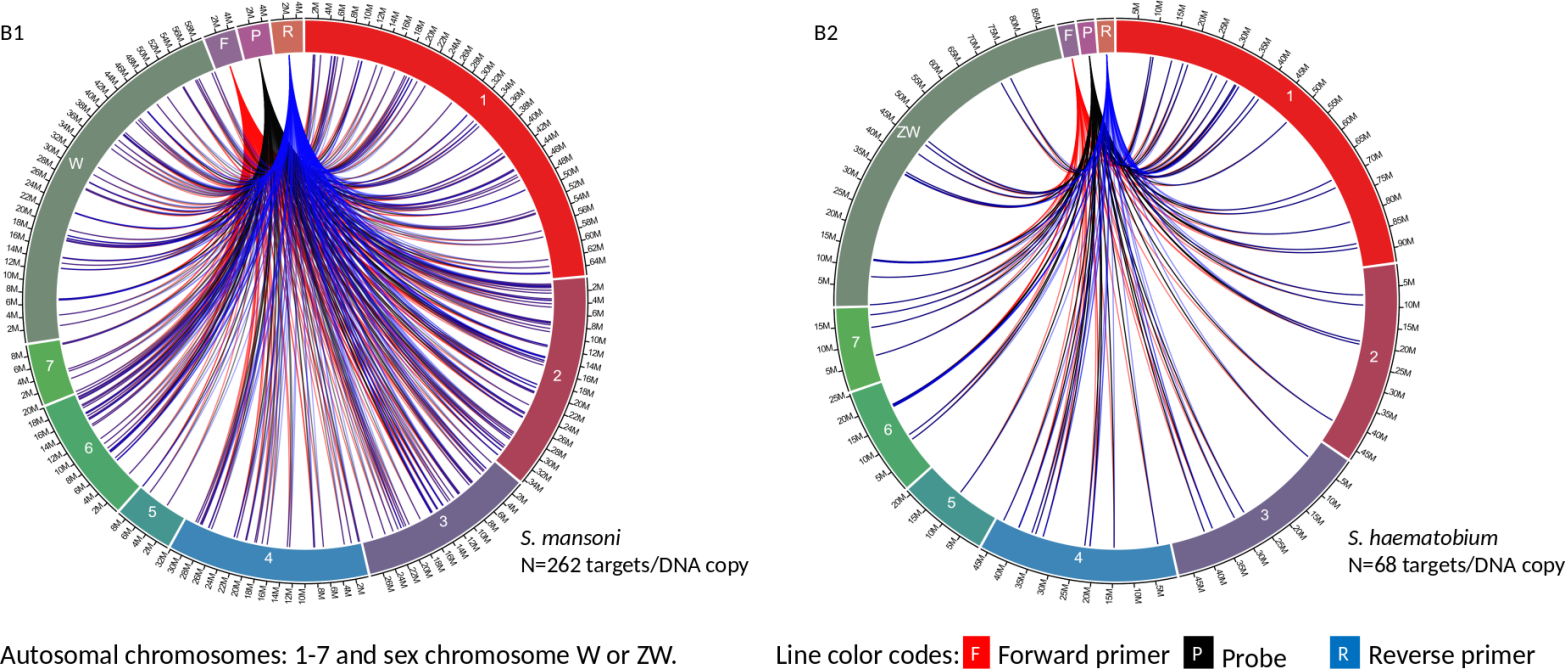
The circa plot for the distribution of IMRS primers and probe on schistosomes. **(B1, B2)** shows the distribution of forward primer 2, reverse primer 2 and probe sequence *S. mansoni* and *S. haematobium*, respectively. The number outside the chromosomes represents the chromosome size in megabase pair (M).

Circa genomics software (student version) was used to visualize the IMRS primer’s targets and distributions throughout the genome of *S. mansoni* and *S. haematobium* (**Figures 1**, **2**). The IMRS primer and probe sequence were aligned using primer BLAST, freely available at NCBI, and the search was limited to no mismatch, and less than 1000 base pair (bp) amplicon size. Primer’s coordinates and their respective chromosomes were copied to an Excel file, and data was converted to comma separated values (CSV) file and then visualized using circa plot software (Gumroad, San Francisco, United States).

### Conventional PCR for *S. haematobium*-16S rRNA

The primers for amplifying 143 bp of *S. haematobium*-16S rRNA was previously highlighted by Alzaylaee et al. (2020). The 25 µL reaction mixture contained 3 µL of DNA, 12.5 µL of One*Taq®* 2X Master Mix with Standard Buffer (New England Biolabs, Massachusetts, Unites States), and 400 nM of forward primer and reverse primer (Macrogen, Massachusetts, Unites States). The thermocycling conditions consisted of initial denaturation at 95°C for 5 minutes, denaturation at 95°C for 30 seconds, annealing at 54°C for 30 seconds, elongation at 72°C for 20 seconds, and final hold at 4°C. PCR products were visualized on 1.5% agarose gel after electrophoresis.

### IMRS conventional PCR

The 25 µL reaction mixture contained 3 µL of DNA, 12.5 µL of One*Taq®* 2X Master Mix with Standard Buffer (New England Biolabs, Massachusetts, Unites States), and 400 nM of forward primer-1 and reverse primer-1 (Macrogen, Massachusetts, Unites States). The thermocycling conditions consisted of initial denaturation at 95°C for 5 minutes, denaturation at 95°C for 30 seconds, annealing at 58°C for 30 seconds, elongation at 72°C for 20 seconds, and final hold at 4°C. PCR products were visualized on 1.5% agarose gel after electrophoresis.

### TaqMan probe-based IMRS qPCR

The 20 µL reaction mix contained 3 µL DNA, 10 µL Luna^®^ Universal Probe qPCR Master Mix (New England Biolabs, Massachusetts, Unites States), 400 nM of forward primer-2 and reverse primer-2, and 200 nM of the FAM/BHQ – labelled probe (Macrogen, Massachusetts, Unites States). The fast-cycling condition and time for qPCR for the three runs are indicated in **Table 1**. The reproducibility was tested for the qPCR run 3 in duplicates.

**Table 1:** The fast-cycling conditions for the simultaneous detection of *S. mansoni* and *S. haematobium* using IMRS qPCR.

	Initial denaturation	Denaturation	Annealing and elongation	Cycles	Time taken
Run 1	95°C for 2 min	95°C for 15 sec	60°C for 20 sec	40	47 min
Run 2	95°C for 2 min	95°C for 10 sec	60°C for 15 sec	40	41 min
Run 3	95°C for 2 min	95°C for 8 sec	60°C for 10 sec	39	36 min

### The lower limit of detection

The standard DNA from *S. mansoni* and *S. haematobium* was diluted to 10 ng/µL, aliquoted, and stored at -80°C. For determining the LLOD, the standard DNA was 10-fold serially diluted from 10 ng/µL to 0.00001 ng/µL. Conventional PCR targeting 143 bp of 16S rRNA in *S. haematobium* and IMRS cPCR for *S. mansoni* and *S. haematobium* were performed in quadruplicates, and IMRS qPCR was carried out in triplicates.

### Sensitivity and specificity

The sensitivity of IMRS qPCR was tested using 7 *Schistosoma*-positive and 13 *Schistosoma*-negative snail samples in duplicates, and for the IMRS PCR, 30 *Schistosoma*-positive snail samples and 49 *Schistosoma*-negative snail samples were used. The *in silico* analysis of specificity was checked in various ways. First, 151bp and 101bp target sequences were searched against the nucleotide database, with the BLAST-search tool being limited to exclude *Schistosoma* spp. Primers for PCR and qPCR were also used to conduct *in silico* PCR using the primer-BLAST tool of NCBI and the *in silico* PCR tool in the UCSC genome browser. The minimum perfect match for primers was set to a minimum of 10 perfect nucleotide matches, and the amplification target was set to 4000 bp for *in silico* PCR using the *in silico* PCR tool of the UCSC genome browser, while up to 6 mismatches and amplification of up to 4000 bp were allowed for *in silico* PCR using primer-BLAST. Additionally, we tested the analytical specificity using a positive *A. lumbricoides* sample.

### Detection of miracidia

A single-blinded study was conducted to assess the performance of IMRS qPCR using four *S. mansoni* miracidia DNA samples isolated from pooled human feces. From pooled fecal samples (Kato-Katz confirmed), the parasite’s ova were obtained by filtration through three sieves (350 µM to 60 µM), and the miracidia hatching from ova was triggered by exposure to light for at least 2 hours. Free swimming miracidia were aspirated with some water into pipettes in batches of 10 fecal samples into two cryovials, with up to a total of 500 and 450 miracidia each, and then stored at -20°C. During DNA extraction, the genomic DNA from miracidia was eluted twice for each sample using molecular-grade water, and blinding was conducted by providing two samples with the first and second elution separately, making a total of four unknowns.

### Copy number of amplification targets

The theoretical relationship between the genome size, DNA concentration, and the number of amplification targets was judged using the mathematical formula for calculating dsDNA copy number (Formula 1). The genome size for selected bacteria, nematodes, platyhelminths, and schistosomes were obtained from the literature, while the genomic DNA concentration was based on assumption and kept constant for the selected organisms.

### Data analysis

The mean cycle threshold (ct) and standard deviation for the ct values were calculated using QuantStudio design and analysis desktop software v1.5 (Thermo Fisher Scientific, Massachusetts, United States) regression analysis, and qPCR data visualization using Python programming in a jupiter notebook version 6.5.4.

### Ethical consideration

This study was reviewed and approved by the Mount Kenya University Ethical Review Committee under reference MKU/ISERC/3022, approval date August 2023 and review number 2066. The study utilized preserved samples, and hence, no informed consent was required.

## Results

### The IMRS primers and probe

The 151 bp IMRS region 5’-AAGGTCGGATCTACAACGCGTCGGTGAGAGCAGTTTTGCTCTATGCTTGTGAAACCTGGCCTCTCCGAGTTGAGGATGTTAGACGTCTCTCTGTGTTCGATCATCGTTGTCTCCGAAGGATTGCTGACATCCAGTGGCAACACCATGTTAG-3’ was used for further primer design customization. The designed IMRS primers, probes sequences are distributed across *S. mansoni* (**Figures 1A1** and **2B1**) and *S. haematobium* genomes (**Figures 1A2**, **2B2**). BLAST-based *in silico* analysis of Refseq representative genomes of schistosomes, indicated that IMRS primers and probe were able to detect all schistosomes of medical importance except *S. japonicum.* Briefly, the IMRS primer pair for IMRS conventional PCR had 215 and 118 amplification targets in *S. mansoni* (taxid:6183) and *S. haematobium* (taxid:6185) respectively, while the IMRS primer pair and probe for IMRS qPCR had 262 and 68 amplification targets in *S. mansoni* and *S. haematobium* genomes, respectively. Further analysis of IMRS targets indicated that many targets for IMRS conventional PCR were 151 bp for both *S. mansoni* and *S. haematobium* (**Figures A1, A2**). These findings suggest that targeted amplification will generate a single PCR band of 151 bp and IMRS conventional PCR can be targeted for the simultaneous detection of *S. mansoni* and *S. haematobium*. The targeting of multiple regions in a single genome is expected to improve sensitivity in IMRS-based PCR and qPCR. Additionally, having an expected band in conventional PCR and a probe sequence in qPCR further increases the confidence in the specificity.

### Sensitivity and LLOD of IMRS conventional PCR

To assess the sensitivity and LLOD of the IMRS-based PCR, 30 *Schistosoma*-positive and 49 *Schistosoma*-negative snail samples and 10-fold serially diluted standard DNA (NR-28910 and NR-31682) were used for PCR. The IMRS-based PCR detected up to 0.0001 ng/µL of 10-fold-serially diluted standard DNA from *S. mansoni* and *S. haematobium* (**Supplementary Figure 1**). The assay offered ten times better analytical sensitivity than conventional PCR, targeting 143 bp in *S. haematobium*, which could only detect up to 0.001 ng/µL (**Supplementary Figure 2**). When tested with the field snail samples, the assay was positive for all 30 *Schistosoma*-positive snail samples (**Supplementary Figure 3**), while 3/49 *Schistosoma*-negative snail samples turned positive by IMRS PCR (**Supplementary Figures 4** and **5**).

The IMRS PCR was also positive when tested against two *Schistosoma* positive baboon’s stool samples (**Supplementary Figure 6**). Thus, the IMRS PCR could detect all stool exam (Kato-Katz technique) confirmed *Schistosoma*-positive samples from baboons and snail samples. With 100% confidence, the detection limit was detected at 0.0001 ng/µL for both *S. mansoni* and *S. haematobium* (**Supplementary Figures 7** and **8**), suggesting that the IMRS PCR has potential to offer better sensitivity for the two major schistosomes of medical importance.

### The Fast-Cycling IMRS qPCR

**Figure 3:**
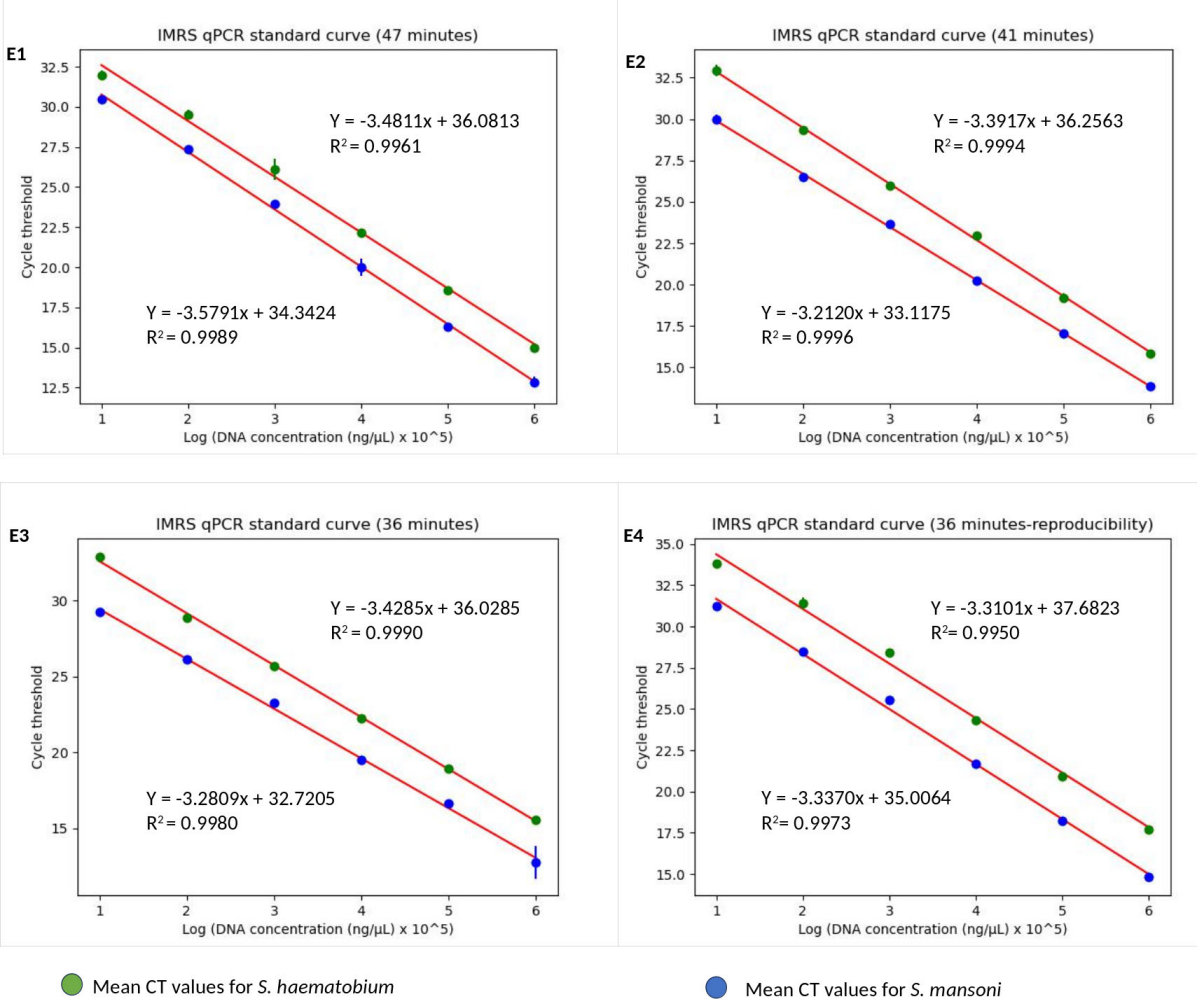
The IMRS qPCR standard curve for the simultaneous detection of *S. mansoni* and *S. haematobium.* **(E1-E4)** are for 47, 41, 36 minutes runs in triplicates, and 36 minutes run-reproducibility, respectively. The regression analysis was performed using python programming.

Next, we aimed to lower the IMRS qPCR amplification time, and therefore three different fast-cycling IMRS qPCR runs were conducted by changing the denaturation, annealing time, and number of qPCR cycles from 40 to 39 (**Table 1**). The regression analysis using python programming showed no statistical differences when the IMRS qPCR was run for 47, 41, and 36 minutes using fast cycling conditions (**Figure 3**, **Supplementary Excel Files 1-4**). In both cases, the IMRS qPCR detected the lowest dilution (0.0001 ng/µL) in reference to the NTC, which gave late ct values. Thus, the IMRS qPCR can be performed in 36 minutes.

### Sensitivity and LLOD of IMRS qPCR

To assess the potential of IMRS-based qPCR to detect *Schistosoma* DNA in 36 minutes, 7 *Schistosoma*-positive snail samples, and 13 *Schistosoma* negative samples (3 were IMRS-PCR positive) and 10-fold serially diluted standard DNA were used for qPCR in duplicate and triplicates, respectively. Like IMRS PCR, the IMRS qPCR detected the lowest dilution of 0.0001 ng/µL, all *Schistosoma* positive samples turned positive, and all *Schistosoma* negative snail samples were negative except three samples that were positive for IMRS PCR (**Supplementary Figure 9**, **Supplementary Excel Files 5 and 6**). This means that some samples which are negative by microscopy and conventional PCR might turn positive when tested by highly sensitive assays. In this case for example, the three negative samples, which turned positive by IMRS PCR turned positive when tested by IMRS qPCR.

### Specificity IMRS PCR and IMRS qPCR

The BLAST results indicated no significant similarity between nucleotide sequences on the database, with 151 bp and 101 bp, except for schistosomes. Further analysis indicated that the sequences were not similar to one schistosome of medical importance, *S. japonicum*. The *in silico* PCR using primer-BLAST indicated no amplification for Olive baboon (taxid:9555), Biomphalaria (taxid:6525), nematode (taxid:6239), *Necator americanus* (taxid:51031), hookworms (taxid:33277), Southeast Asian liver fluke (taxid:6198), Strongyloididae (taxid:6246), Filariidae (taxid:6296), Loa (taxid:7208), Onchocercidae (taxid:6296), Taeniidae (taxid:6208), Echinococcus (taxid:6209), *Echinococcus granulosus* (taxid:6210), and Cestoda (taxid:6199).

The *in silico* PCR using UCSC genome browser, also, indicated no possibility for PCR to occur for the followings: Flat worms (*Echinococcus granulosus*, *Macrostomum lignano*, and *S. japonicum*), Nematodes (*Necator americanus*, *Trichinella spiralis*, *Strongyloides ratti*, *Oscheius tipulae*, P*anagrolaimus* sp., *Caenorhabditis briggsae*, *C. inopinata*, *C. remanei*, and *C. tropicalis*), Trypanasomes (*Trypanosoma brucei brucei*, *T. brucei gambiense*, *T. congolense*, *T. conorhini*, *T. cruzi*, *T. grayi*, *T. rangeli*, *T. theileri*), Leishmania (*Leishmania braziliensis*, *L. donovani*, *L. infantum*, *L. major strain Friedlin*, *L. mexicana*, *L. panamensis*, *L. tarentolae*, and *L. pyrrhocoris*), Malaria parasites (*P. falciparum*, and *P. vivax*) and human genome *(Homo sapiens)*. Herein, S*. japonicum*, which indicated no chance for amplification with IMRS primer, based on BLAST results, showed no amplification with *in silico* PCR using UCSC genome browser, and the *A. lumbricoides*, which was tested experimentally in the laboratory, indicated no amplification using IMRS primers and probe.

### Concentration-based detection of miracidia DNA

The blinded study was conducted using four unknown samples from two pooled samples, each eluted twice during DNA extraction. For the first elution from each sample, the DNA concentrations were relatively higher compared to the second elution, which caused the variation in mean ct values of qPCR results. The unknown samples from the first elution resulted in early mean ct values of 14.1 and 14.2 for 500 and 450 miracidia samples, respectively. On the other hand, the second elution from each sample resulted in a slight reduction of DNA concentration, and hence, the mean ct value increased to 15.2 for the two samples. The standard *S. mansoni* DNA sample (10 ng/µL) amplified with mean ct values of 13.1, while the NTC sample gave a late ct value of 34 (**Supplementary Excel File 6**).

### The genome size and number of amplification targets are inversely proportional

Based on the genome of selected organisms, the increase in genome size causes a reduction in the number of amplification targets, given a constant DNA concentration (**Table 2**). This means that when there is too low of a DNA concentration, the chances are low for NAATs to detect a single genome target from organisms with large genomes compared to organisms with small genome sizes.

**Table 2:** The relationship between genome size and genome copy number.

N/a	Genome size (Mb)	Amount (ng)	Genome copy number	References
Bacteria	0.1-16	0.001	57.9-9264	(Rodríguez-Gijón et al., 2022)
nematodes	42-700	0.001	1.32-22.05	(International Helminth Genomes Consortium, 2019)
platyhelminths	104-1259	0.001	0.74-8.91	(International Helminth Genomes Consortium, 2019)
*S. mansoni*	409.57	0.001	2.26	(Stroehlein et al., 2022)
*S. haematobium*	400.27	0.001	2.31	(Stroehlein et al., 2022)

## Discussion

Herein, we describe a rapid and sensitive qPCR assay for detecting schistosomes, leveraging on IMRS, and how targeting repeated sequences is essential for parasites with large genomes. Theoretically, the copy number of amplification targets is inversely proportional to the genome size (Formula 1). Hence, large genome sizes can significantly reduce the sensitivity of NAATs, especially when an assay has a single target in a given genome. The use of primers that target identical multi-repeat sequences (IMRS) is a significant way to reduce the impact of low genome copies, and this approach has resulted in an improved sensitivity in the detection of *P. falciparum* using intercalating-dye based real-time PCR (Raju et al., 2019) and isothermal amplification (Kolluri et al., 2021).

The *in silico* validation using NCBI-BLAST and UCSC *in silico* PCR indicated that the designed IMRS primers and probe are specific to the *Schistosoma* genus, being able to detect all schistosomes of medical importance except *S. japonicum* (World Health Organization [WHO], 2022c), and have multiple repeat targets in both *S. mansoni* and *S. haematobium*. These repeat targets were based on amplifiable region limited to less than 1000 bp with no mismatch because for the time-limited amplification, too large amplicons lower the efficiency of NAATs (Van Holm et al., 2021). The IMRS primers chosen for conventional PCR were amplifying complete 151 bp repeat region, whereas the primers chosen for qPCR were amplifying a smaller region of 101 bp within the 151 bp to increase the efficiency of qPCR.

In IMRS conventional PCR, the amplification of approximately 151 bp enabled detection as low as 0.0001 ng/µL of standard genomic DNA from *S. mansoni* and *S. haematobium* and 100% sensitivity for detecting positive *Schistosoma* snail and baboon stool samples. This represents a detection of as little as a single genome copy per microliter and a tenfold improvement over conventional PCR, targeting 143 bp in *S. haematobium.* The findings on the improved sensitivity of IMRS-based NAATs over the conventional approach are supported by other studies (Raju et al., 2019; Kolluri et al., 2021).

For the IMRS qPCR, the aim was to lower the amplification time to at most 40 minutes, as opposed to most standard qPCR assays, which takes up to 1 hour on average (Nayab et al., 2008; Bustin and Nolan, 2020). To attain abovementioned objective, the fast-cycling conditions were applied as described by Milosevic et al. (2021). In their study, however, Milosevic et al. (2021) managed to develop an ultra-sensitive reverse transcriptase (RT) qPCR, which could detect as little as 25 copies of SARS-CoV-2 RNA genome in 30 minutes. For our study, the qPCR detected as little as one genome copy per microliter of *Schistosoma* genome within 36 minutes. The difference in polymerase enzyme used accounts for six minutes amplification time difference. The SpeExcelTAR HS DNA Polymerase used by Milosevic et al. is optimized for a 10s/kb extension rate, while the standard DNA polymerase has a 60s/kb extension rate (Milosevic et al., 2021). Reducing the amplification time in qPCR will enable processing of many samples in a reasonable time, especially for surveillance studies.

**Figure 4:**
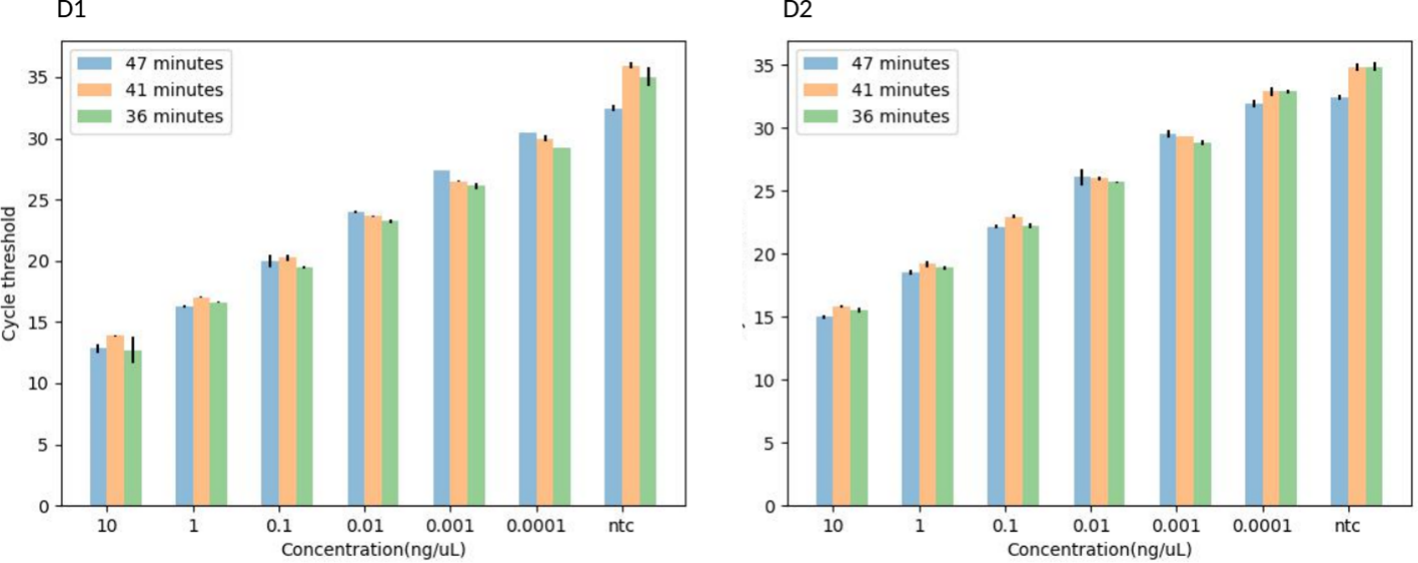
A histogram showing the TaqMan-probe based-IMRS qPCR for the simultaneous detection of *S. mansoni* **(D1)** and *S. haematobium* **(D2)** in 47, 41, and 36 minutes. Copy number = (Amount of DNA (ng) × Avogadro's constant) / (Length (bp) × Conversion factor × Average mass of 1 bp of dsDNA).

The 36 minutes amplification time was based on the regression analysis, whereby there were no significant differences in amplification efficiency for the qPCR which took 47, 41 and 36 minutes, all giving R2 > 0.99 (**Figure 3**), an acceptable amplification efficiency (90-110%), and as low as 0.0001 ng/µL was detected. When tested with field samples, the IMRS qPCR was positive for all positive *Schistosoma* snail samples, and negative *Schistosoma* snail samples that were positive for IMRS PCR turned positive with IMRS qPCR (**Supplementary Figure 9**). This indicated agreement between IMRS PCR and IMRS qPCR. In most cases, however, the no template control (NTC) gave late ct value caused by background fluorescence (**Figure 4**), and therefore, for reliable results, samples must be run in triplicates, and the NTC must be included to rule out positive and negative samples (Keller et al., 2020; Forootan et al., 2017).

The reported false negatives when NAATs are used to detect schistosomes from human stool and urine (Hoekstra et al., 2022; Meurs et al., 2015) might have been caused by the large genome size, which significantly reduces the copy number of amplification targets, and hence targeting many identical repeats should enhance sensitivity much better than targeting single to very few targets. Therefore, the ability of IMRS PCR and IMRS qPCR to amplify as little as one genome copy per microliter of standard DNA for both *S. mansoni* and *S. haematobium* standard DNA, all *Schistosoma* positive baboon’s stool and snail samples indicates the potential of IMRS primers to diagnose schistosomiasis caused by the two major species, *S. mansoni* and *S. haematobium* as well as individual with low parasite levels. Additionally, the IMRS primers detected 3/49 *Schistosoma* samples, previously confirmed negative by microscopy and cPCR that target 500 bp of the internal transcribed spacer region (ITS1) in schistosomes and 600 bp for snail DNA. This might indicate that ITS1 PCR might have missed some *Schistosoma*-positive samples.

The LLOD for IMRS PCR and qPCR for *S. mansoni* and *S. haematobium* was comparable to much higher than genus-specific LAMP assay for schistosomes that used standard DNA from BEI resources, whereby the authors reported detection limit 0.01 ng/µL, 0.001 ng/µL, 0.001 ng/µL and 0.0001 ng/µL for the detection of *S. bovis*, *S. mansoni*, *S. intercalatum* and *S. haematobium*, respectively (Fernández-Soto et al., 2020). For most studies that used control DNA extracted from in-house schistosomes, authors reported varying analytical sensitivity, with the detection limit ranging from 0.1 ng/µL to 0.0000001 ng/µL (García-Bernalt Diego et al., 2021; Gandasegui et al., 2015; Crego-Vicente et al., 2021; Abbasi et al., 2010; Kumagai et al., 2010; Rostron et al., 2019). This suggests that more research is needed to compare how well NAATs work in a single controlled setting. For instance, Gandasegui et al. (2015) reported 10 times better sensitivity using *Schistosoma* DNA from urine samples compared to the *Schistosoma* positive control from BEI resources, which further show how the DNA control might influence the LLOD estimates, and necessitate the WHO’s call for more data on sensitivity and specificity of NAATs (World Health Organization [WHO], 2022b).

This study has some limitations. First, the study doesn’t have *S. mansoni* stool samples to compare the IMRS tests with KK and Sm 1–7 PCR, or any human S. *haematobium* urine samples to compare the tests with Dra1 PCR and urine examination. Additionally, the *in silico* PCR to confirm the analytical specificity was limited by the genomic data available at primer-BLAST and UCSC genome browser, and only one sample was used to experimentally test the specificity of IMRS primers. Nevertheless, to the best level of our knowledge, the study reports the first rapid, and probe-based IMRS qPCR for schistosomes, and the findings presented might positively impact the development of sensitive assays for detecting parasites, particularly platyhelminths, whose genomes are large, and have many repeated sequences (International Helminth Genomes Consortium, 2019).

## Conclusion and recommendation

Our findings indicate that IMRS primers offer an improved sensitivity than conventional primers and microscopy, therefore the IMRS primers could be used when an improved sensitivity is a primary requirement, especially for parasites with large genomes. Nevertheless, further studies are required to assess the specificity and sensitivity of IMRS-based diagnostics compared to other diagnostic approaches, including qPCR methods, for *Schistosoma* spp. and *Schistosoma*-related species. These studies should include statistically significant human samples from areas with both high and low prevalence of circulating single and multiple *Schistosoma* species. Additionally, the assessment should encompass multiple sample types, such as stool, urine, blood serum, dried blood samples, and saliva.

## Data availability statement

The original contributions presented in the study are included in the article/**Supplementary Material**. Further inquiries can be directed to the corresponding authors.

## Ethics statement

The studies involving humans were approved by Mount Kenya University Ethical Review Committee under reference MKU/ISERC/3022, approval date August 2023 and review number 2066. The studies were conducted in accordance with the local legislation and institutional requirements. The human samples used in this study were acquired from gifted from another research group. Written informed consent for participation was not required from the participants or the participants’ legal guardians/next of kin in accordance with the national legislation and institutional requirements. The animal study was approved by Mount Kenya University Ethical Review Committee under reference MKU/ISERC/3022, approval date August 2023 and review number 2066. The study was conducted in accordance with the local legislation and institutional requirements.

## Author contributions

OA: Conceptualization, Data curation, Formal analysis, Investigation, Methodology, Software, Validation, Visualization, Writing – original draft, Writing – review & editing. BK: Conceptualization, Methodology, Resources, Validation, Writing – review & editing. SK: Conceptualization, Software, Writing – review & editing. CS: Conceptualization, Methodology, Resources, Validation, Writing – review & editing. EN: Resources, Validation, Writing – review & editing. MO: Resources, Validation, Writing – review & editing. SN: Conceptualization, Funding acquisition, Methodology, Supervision, Validation, Writing – review & editing. CKK: Resources, Writing – review & editing. GM: Conceptualization, Methodology, Writing – review & editing. LO: Conceptualization, Funding acquisition, Methodology, Resources, Supervision, Validation, Writing – review & editing. SL: Conceptualization, Software, Writing – review & editing. JG: Conceptualization, Funding acquisition, Methodology, Resources, Supervision, Validation, Writing – review & editing.
