# Supplementary material for: Development of a rapid and highly sensitive nucleic acid-based diagnostic test for schistosomes, leveraging on identical multi-repeat sequences
Source: Front Parasitol. 2024 Mar 14;3:1361493. doi: 10.3389/fpara.2024.1361493 (PMC11731689; doi:10.3389/fpara.2024.1361493)
Supplement: Supplementary file 1 [file DataSheet_1.pdf]

SUPPLEMENTARY FIGURES/ TABLES

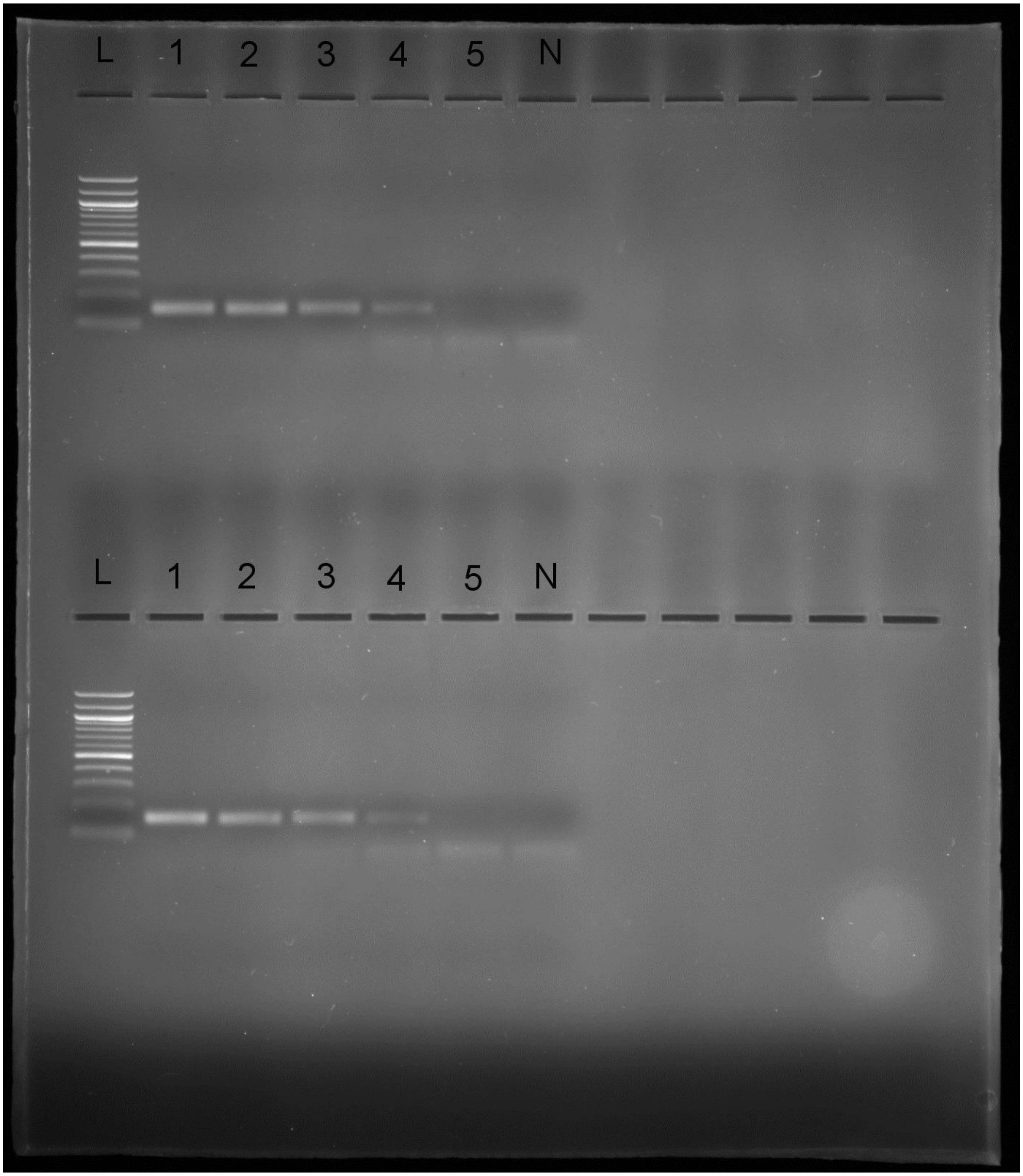

Supplement Figure 1: PCR verification of expected ~151 bp target length amplicon. Lanes 1-5; 10-fold serially dilutions of *S. mansoni* (top) and *S. haematobium* (bottom) standard DNA (ATCC) from 0.1 ng/µL to 0.00001 ng/µL. N and L represents template control and 100 bp ladder, respectively.

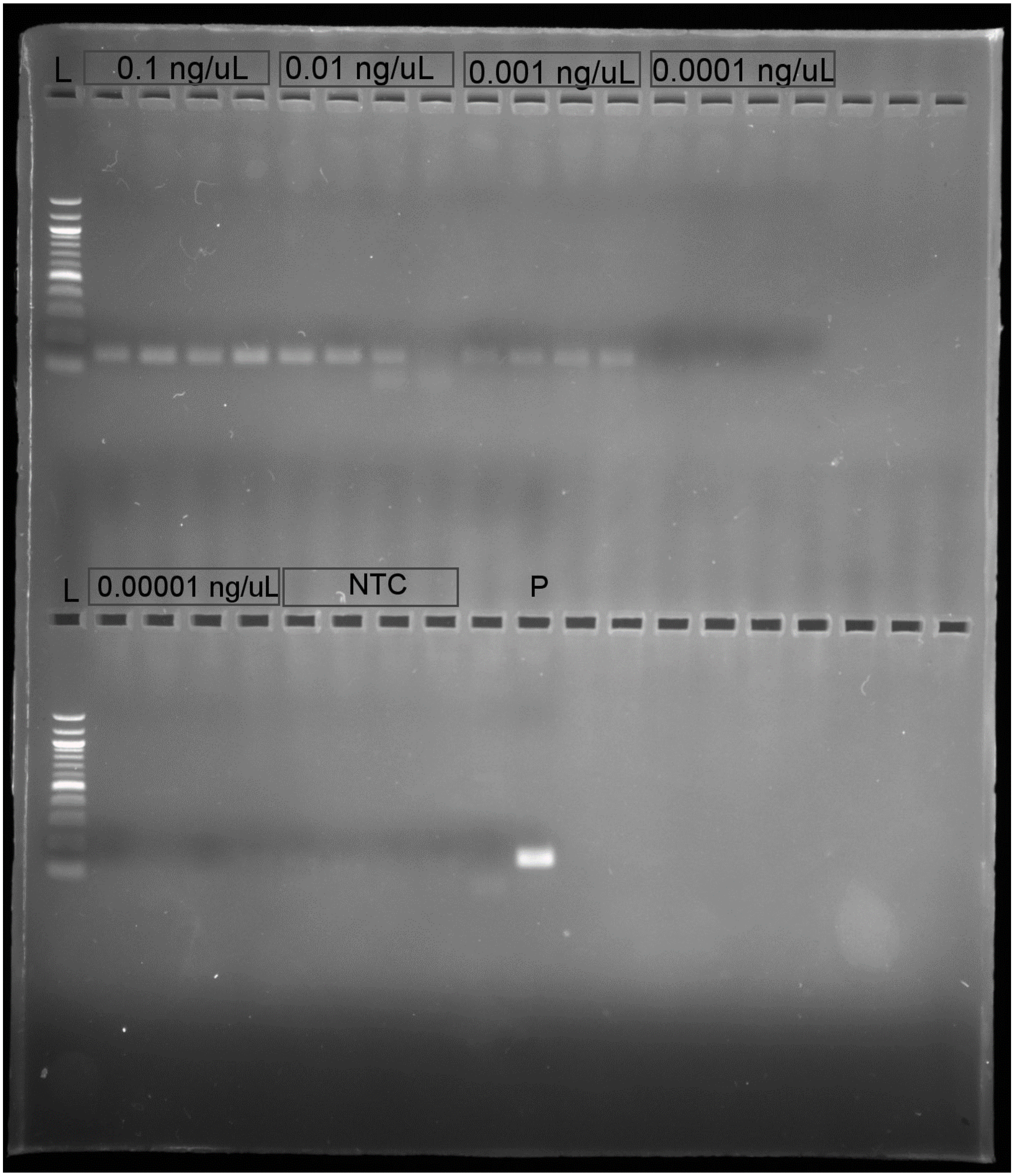

Supplement Figure 2: The 16s RNA PCR verification of expected ~143 bp target length amplicon using *S. haematobium* standard DNA in quadruplicates. The DNA was 10-fold serially diluted from 0.1 ng/ µL to 0.00001 ng/ µL. NTC and P stands for no template control and positive control, respectively. L stand for 100 bp ladder.

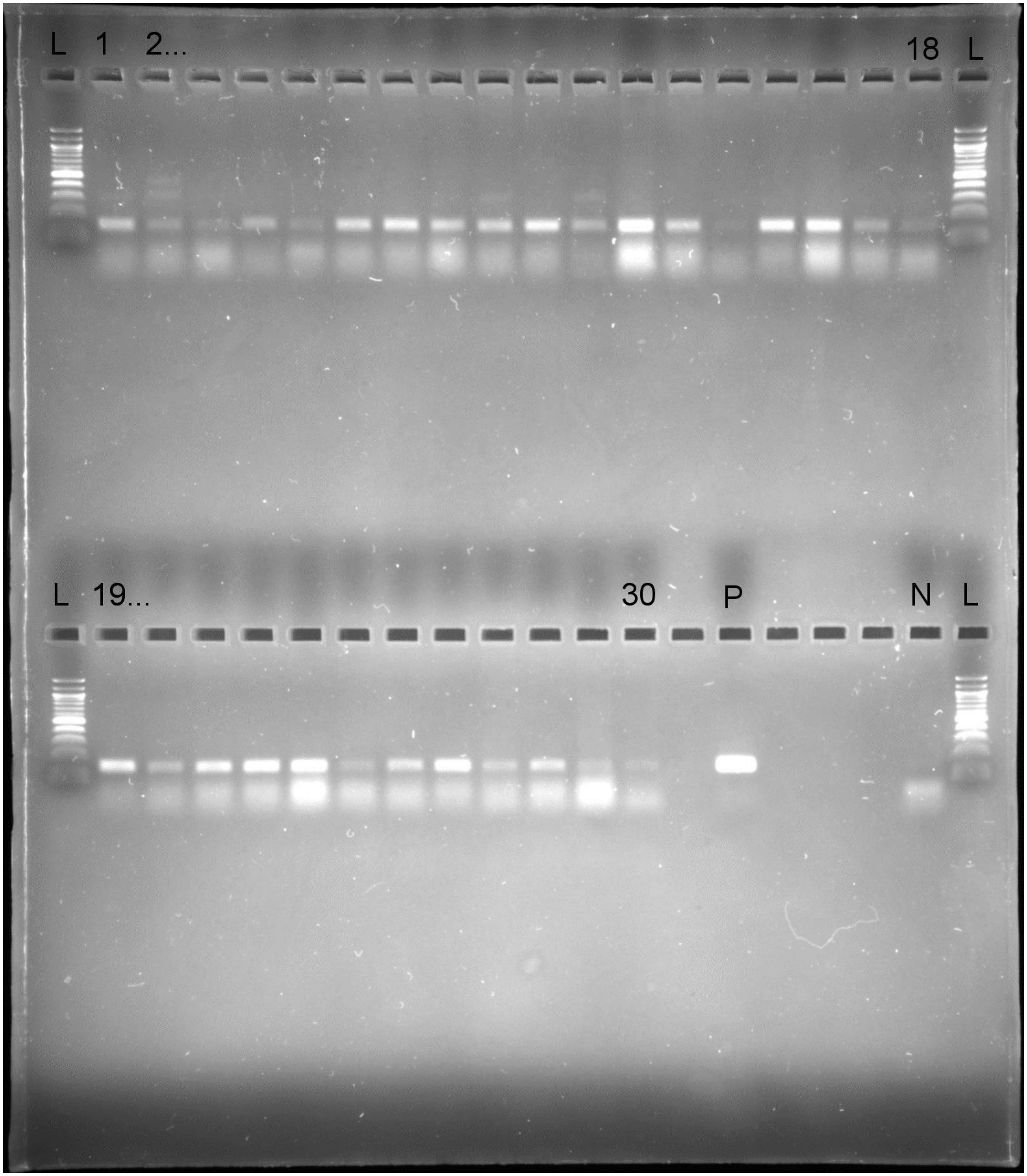

Supplement Figure 3: The IMRC PCR for 30 Schistosoma positive snail samples. Samples were positive by microscopy, and all turned positive by IMRS PCR. N and P stands for no template control and positive control, respectively. L stand for 100 bp ladder.

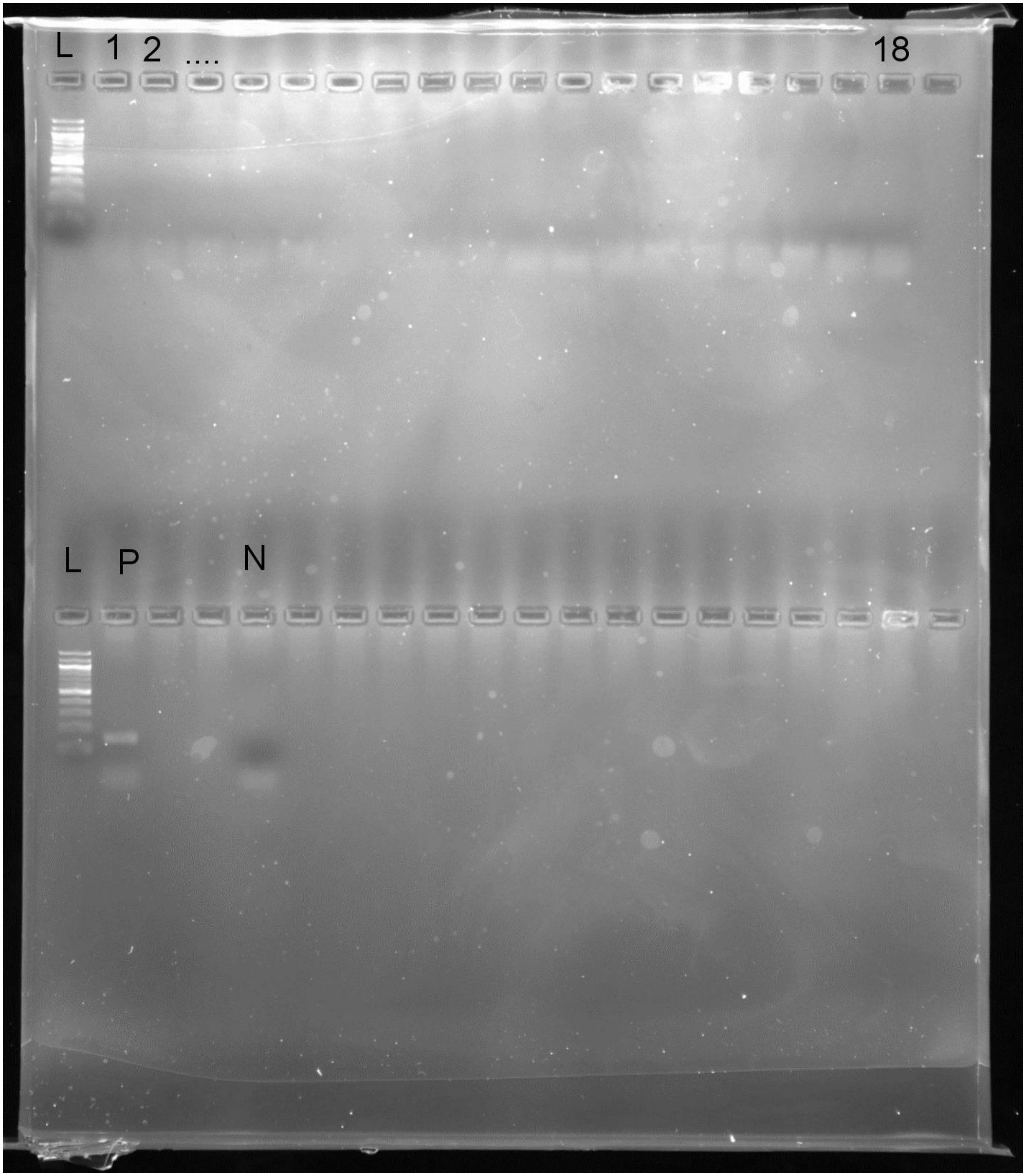

Supplement Figure 4: IMRS PCR for 18 Schistosoma negative snail samples. N and P stands for no template control and positive control, respectively. L stand for 100 bp ladder.

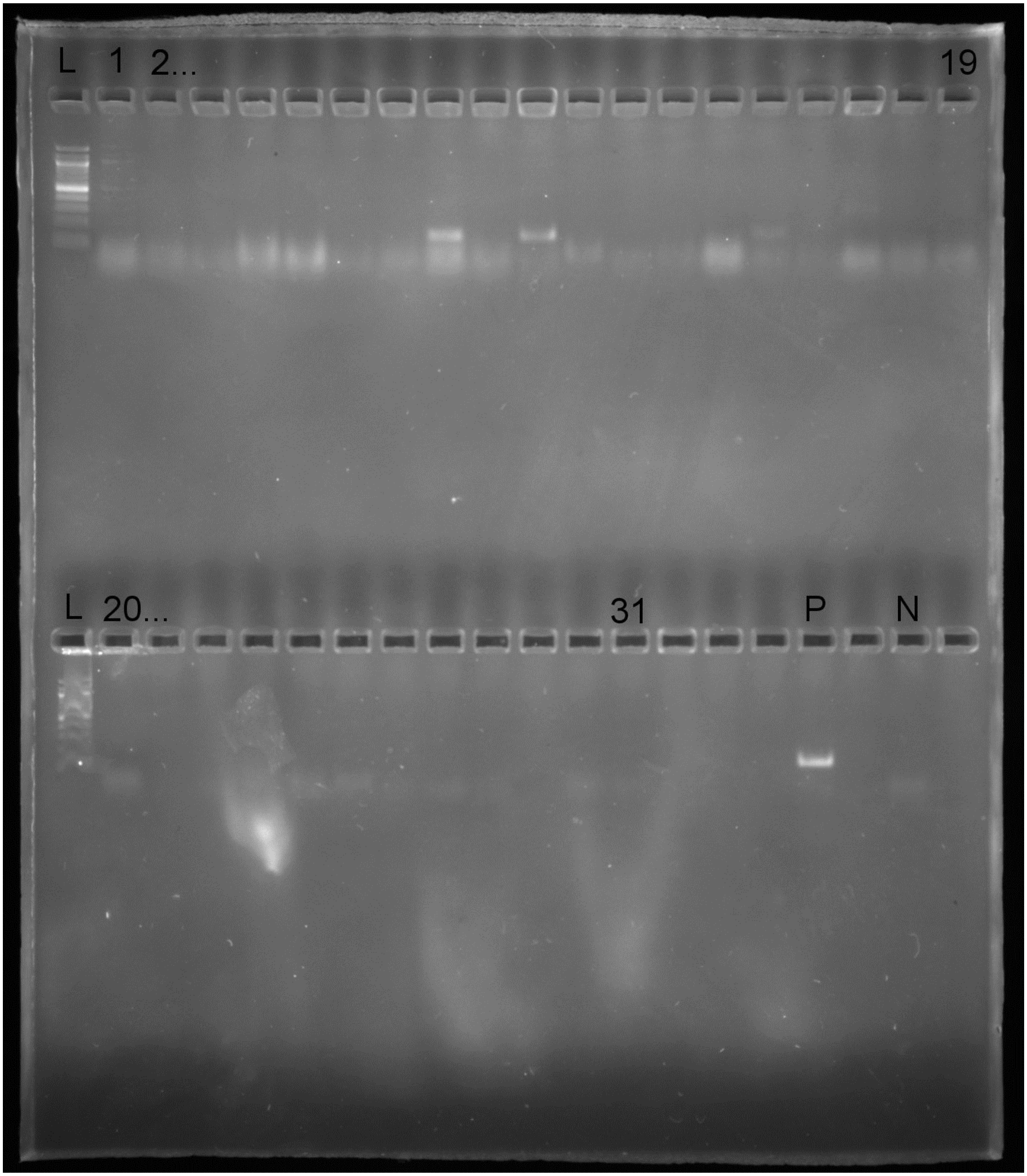

Supplement Figure 5: IMRS PCR for 31 Schistosoma negative snail samples. Three samples, which confirmed negative by microscopy and conventional PCR turned positive when tested by IMRS PCR. N and P stands for no template control and positive control, respectively. L stand for 100 bp ladder.

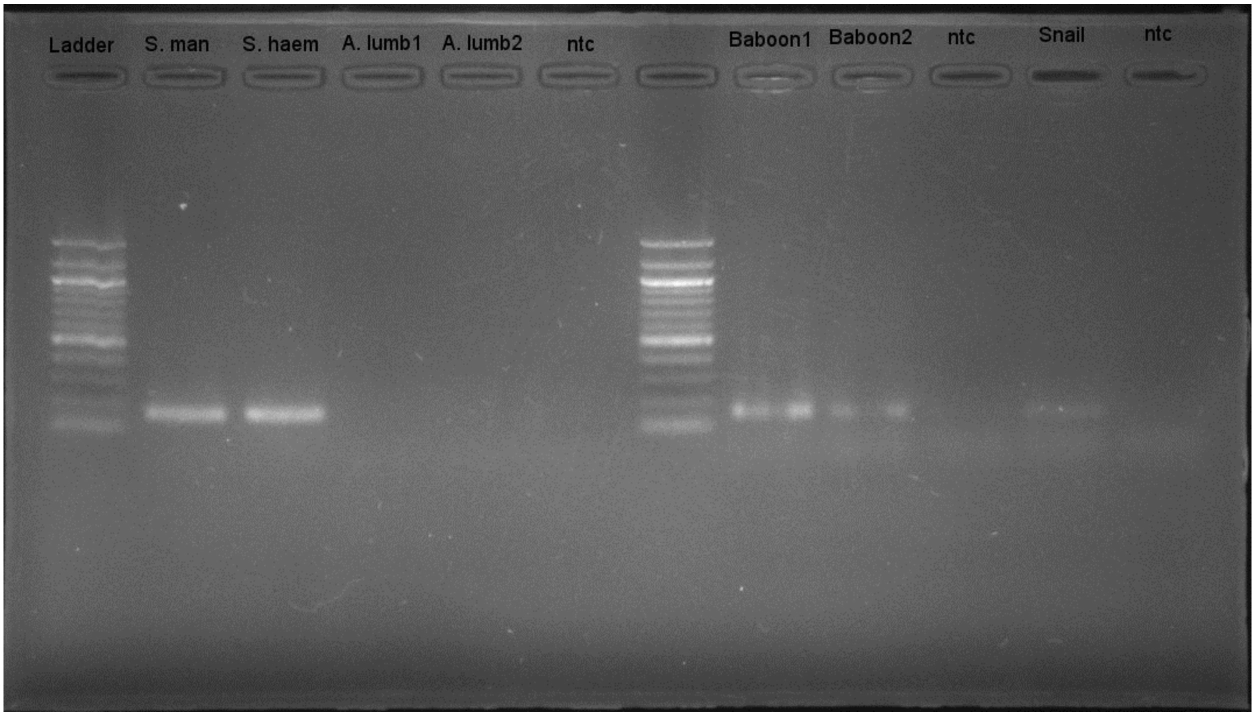

Supplementary Figure 6; The IMRS PCR for *S. mansoni* and *S. haematobium* standard DNA (BEI resources), two *A. lumbricoides* positive DNA samples, two *S. mansoni* baboon samples, and one *S. mansoni* snail sample.

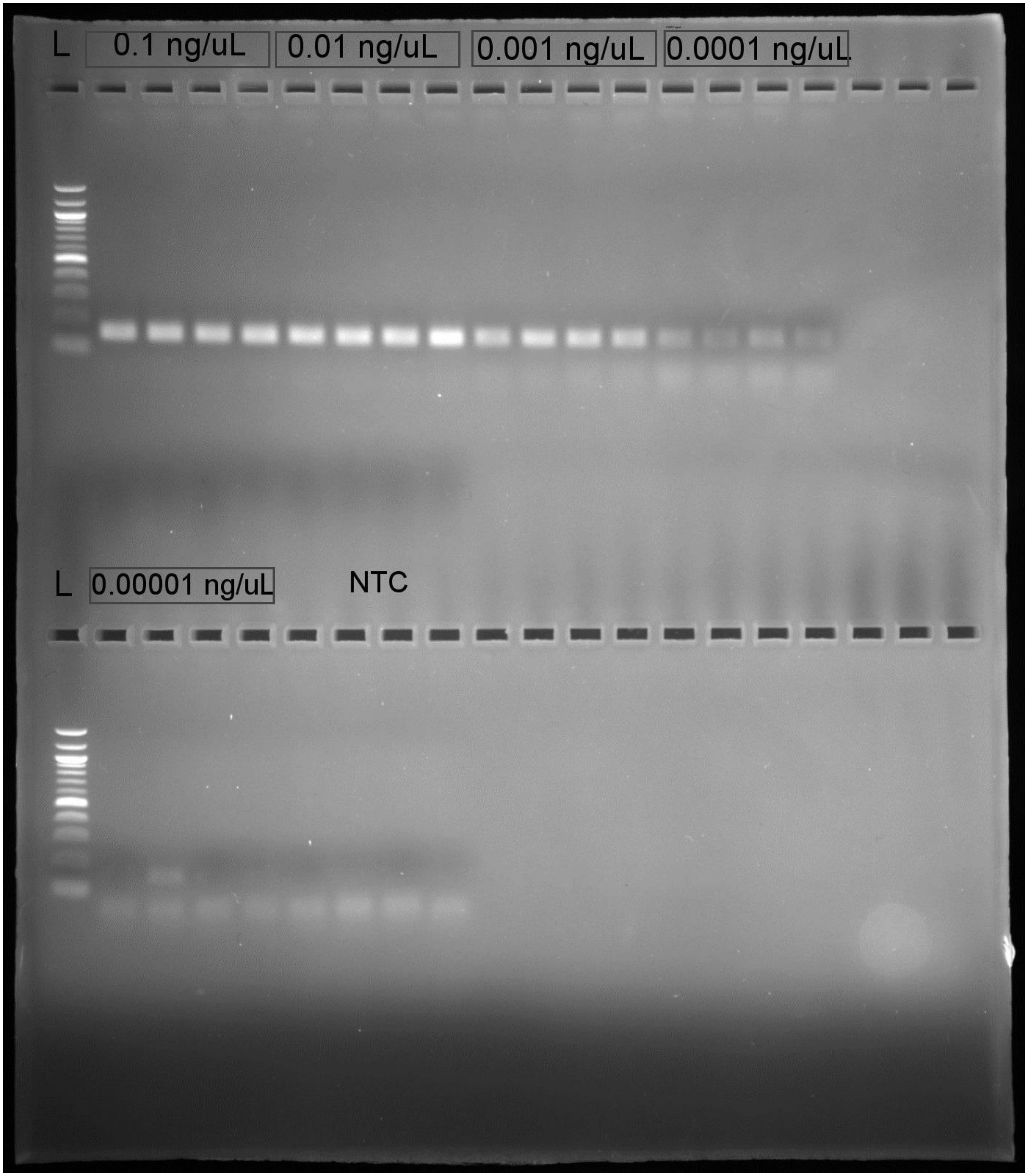

Supplement Figure 7: IMRS PCR verification of expected ~151 bp target length amplicon using *S. mansoni* standard DNA in quadruplicates. The DNA was 10-fold serially diluted from 0.1 ng/µL to 0.00001 ng/µL. NTC and L stand for no template control and 100 bp ladder, respectively.

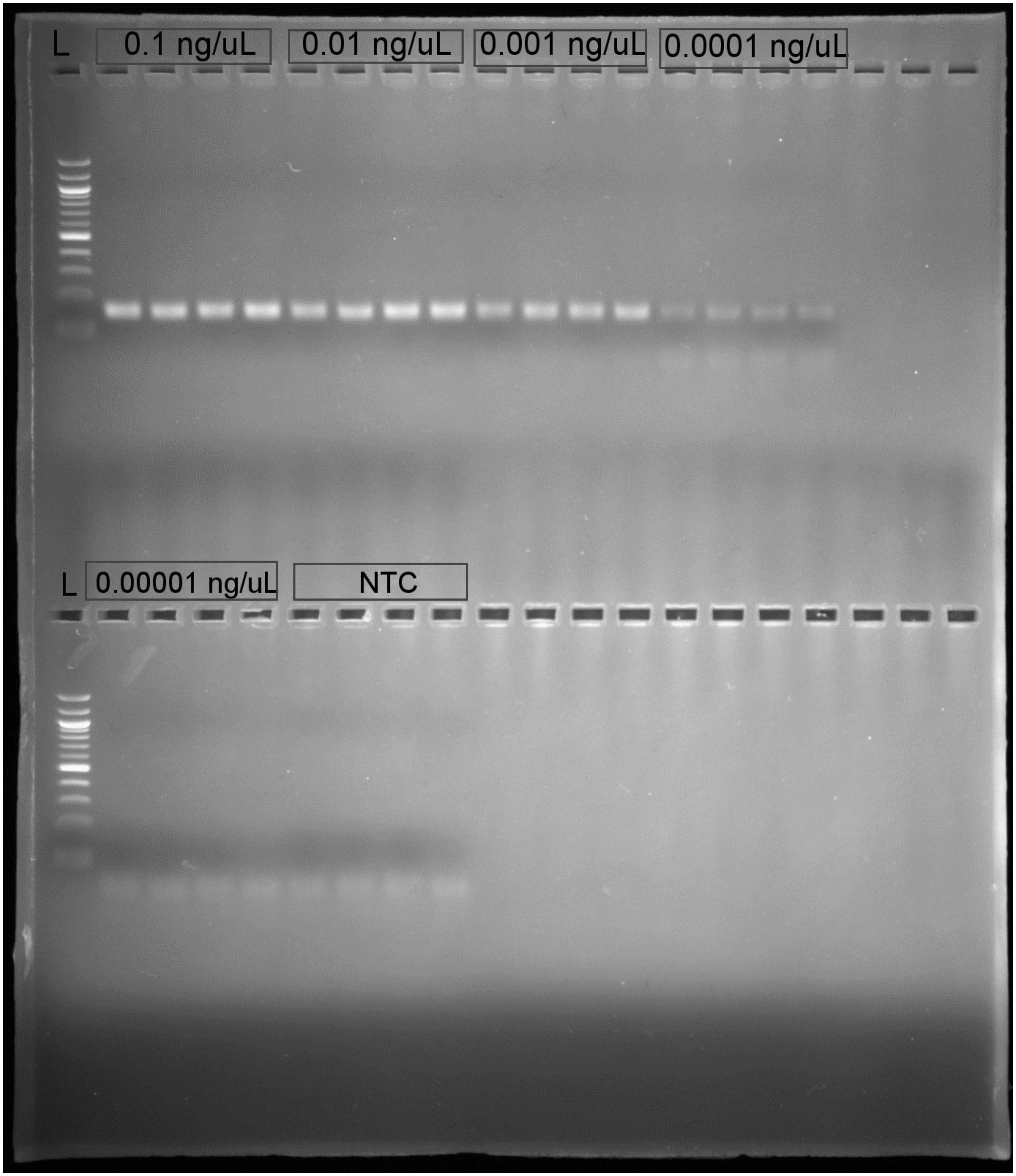

Supplement Figure 8: IMRS PCR verification of expected ~151 bp target length amplicon using *S. haematobium* standard DNA in quadruplicates. The DNA was 10-fold serially diluted from 0.1 ng/µL to 0.00001 ng/µL. NTC and L stand for no template control and 100 bp ladder, respectively.

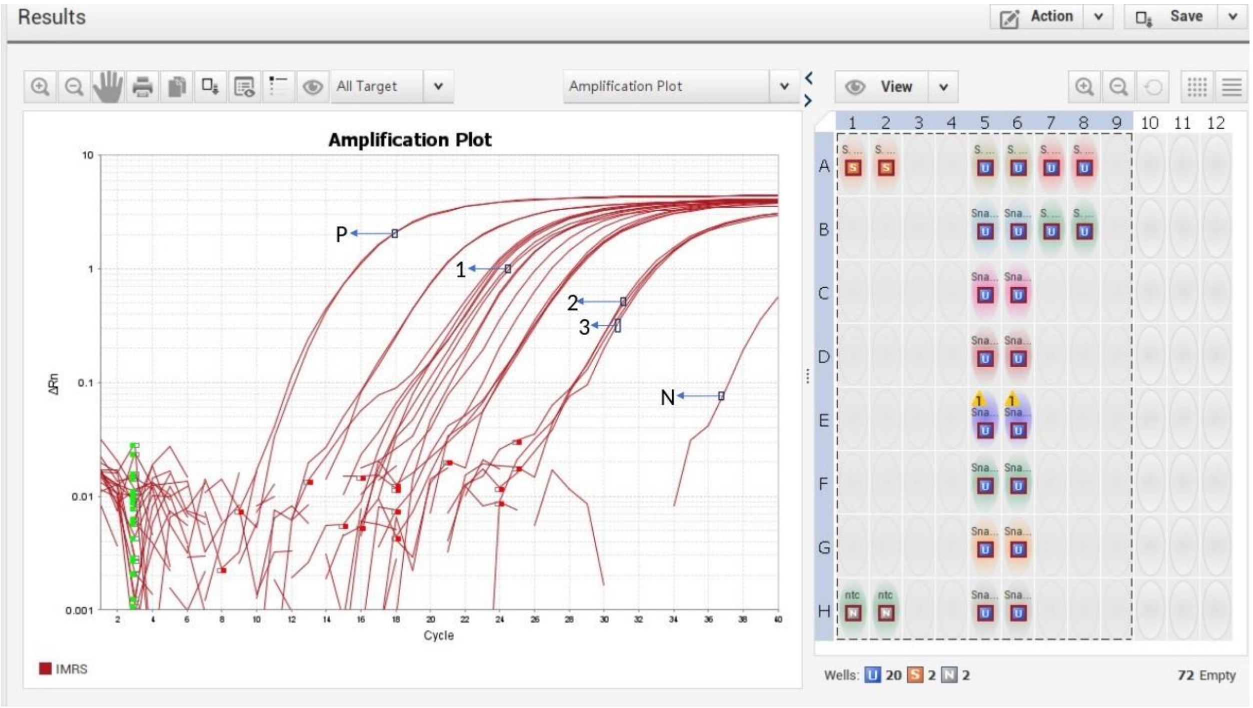

Supplement figure 9: The amplification plots for the detection of Schistosoma DNA in duplicates. 1-3 are the three schistosoma-negative conventional PCR confirmed samples but positive for IMRS PCR. P and N are positive and no template control, respectively. The unlabelled amplification plots represent seven Schistosoma-positive snail samples.

Formula 1

$$Copy\ number = \frac{Amount\ of\ DNA\ (ng)\ X\ Avogadro's\ constant}{Length\ (bp)\ X\ Conversion\ factor\ \ X\ average\ mass\ of\ 1\ bp\ of\ dsDNA}$$

Avogadro's constant ($6.022x10^{23}$)

https://www.technologynetworks.com/tn/tools/copynumbercalculator
